# The Circulating Transcriptome as a Source of Biomarkers for Melanoma

**DOI:** 10.3390/cancers11010070

**Published:** 2019-01-10

**Authors:** Carla Solé, Daniela Tramonti, Maike Schramm, Ibai Goicoechea, María Armesto, Luiza I. Hernandez, Lorea Manterola, Marta Fernandez-Mercado, Karmele Mujika, Anna Tuneu, Ane Jaka, Maitena Tellaetxe, Marc R. Friedländer, Xavier Estivill, Paolo Piazza, Pablo L. Ortiz-Romero, Mark R. Middleton, Charles H. Lawrie

**Affiliations:** 1 Molecular Oncology group, Biodonostia Research Institute, San Sebastián 20012, Spain; carla.sole@biodonostia.org (C.S.); m.schramm02@googlemail.com (M.S.); ibai.goicoechea@biodonostia.org (I.G.); maria.armesto@biodonostia.org (M.A.); luiza.hernandez@liu.se (L.I.H.); lorea.manterola@biodonostia.org (L.M.); marta.fernandez@biodonostia.org (M.F.-M.); maitena.tellaeche@biodonostia.org (M.T.); 2 Department of Oncology, University of Oxford, Oxford OX3 9DU, UK; dtramonti@hotmail.com (D.T.); mark.middleton@oncology.ox.ac.uk (M.R.M.); 3 Faculty of Biosciences, University of Heidelberg, Heidelberg 69120, Germany; 4 Onkologikoa-Oncology Institute Gipuzkoa, Gipuzkoa 20012, Spain; kmujika@onkologikoa.org; 5 Department of Dermatology, Hospital Universitario de Donostia, San Sebastian 20012, Spain; annadonosti@gmail.com (A.T.); iratzia@hotmail.com (A.J.); 6 Genomics and Disease group, Centre for Genomic Regulation (CRG), Barcelona 08003, Spain; marc.friedlander@scilifelab.se (M.R.F.); xavier.estivill@crg.es (X.E.); 7 Universitat Pompeu Fabra (UPF), Barcelona 08002, Spain; 8 Centro de Investigación Biomédica en Red Epidemiología y Salud Pública (CIBERESP), Barcelona 08002, Spain; 9 Hospital del Mar Research Institute (IMIM), Barcelona 08003, Spain; 10 Science for Life Laboratory, The Wenner-Gren Institute, Stockholm University, Stockholm SE-106 9, Sweden; 11 Wellcome Trust Centre for Human Genetics, University of Oxford, Oxford OX3 7BN, UK; p.piazza@imperial.ac.uk; 12 Imperial BRC Genomics Facility, Imperial College London, London SW7 2AZ, UK; 13 Department of Dermatology, 12 de Octubre Hospital, Madrid 28041, Spain; pablo.ortiz@salud.madrid.org; 14 Medical School, Universidad Complutense, Institute i+12, Centro de Investigación Biomédica en Red en Oncologia (CIBERONC), Madrid 28040, Spain; 15 Radcliffe Department of Medicine, University of Oxford, Oxford OX3 9DU, UK; 16 IKERBASQUE, Basque Foundation for Science, Bilbao 48013, Spain

**Keywords:** melanoma, plasma, liquid biopsy, miRNA, mRNA, biomarker, YRNA, RNA species

## Abstract

The circulating transcriptome is a valuable source of cancer biomarkers, which, with the exception of microRNAs (miRNAs), remains relatively unexplored. To elucidate which RNAs are present in plasma from melanoma patients and which could be used to distinguish cancer patients from healthy individuals, we used next generation sequencing (NGS), and validation was carried out by qPCR and/or ddPCR. We identified 442 different microRNAs in samples, eleven of which were differentially expressed (*p* < 0.05). Levels of *miR-134-5p* and *miR-320a-3p* were significantly down-regulated (*p* < 0.001) in melanoma samples (*n* = 96) compared to healthy controls (*n* = 28). Differentially expressed protein-encoding mRNA 5′-fragments were enriched for the angiopoietin, p21-activated kinase (PAK), and EIF2 pathways. Levels of *ATM1*, *AMFR*, *SOS1*, and *CD109* gene fragments were up-regulated (*p* < 0.001) in melanoma samples (*n* = 144) compared to healthy controls (*n* = 41) (AUC = 0.825). Over 40% of mapped reads were YRNAs, a class of non-coding RNAs that to date has been little explored. Expression levels of *RNY3P1*, *RNY4P1*, and *RNY4P25* were significantly higher in patients with stage 0 disease than either healthy controls or more advanced stage disease (*p* < 0.001). In conclusion, we have identified a number of novel RNA biomarkers, which, most importantly, we validated in multi-center retrospective and prospective cohorts, suggesting potential diagnostic use of these RNA species.

## 1. Introduction

Although malignant melanoma accounts for ~4% of skin cancer cases, it accounts for ~75% of all associated mortalities. In the US alone, it is estimated there were 87,000 new cases and nearly 10,000 deaths due to melanoma in 2017 [1]. Furthermore, the incidence of melanoma has been increasing at faster rate than any other cancer type, having doubled since 1973 [2]. The clinical case for early diagnosis of melanoma is compelling, as if detected early enough, (stage I) 5 year survival is >95%, whereas in advanced melanoma (stage IV) survival is just 10–20% [3].

Non-invasive diagnostics, or liquid biopsies, represent a major advance towards earlier diagnosis and disease monitoring of cancer patients, including those with melanoma. As a consequence, there has been a great deal of interest in recent years in the potential of circulating nucleic acids, and in particular circulating microRNAs (miRNAs) [4,5]. In melanoma, several studies have implemented circulating miRNA in metastasis and risk of recurrence [6,7]. However, outside of miRNAs there has been little research on other cell-free (cf) RNA species in the circulating transcriptome. Part of the reason for this paucity of knowledge is the presence of high levels of RNase activity in blood, which typically results in fragmentation of longer RNA species such as mRNA [8,9]. This makes detection of these molecules particularly challenging. The advent of next-generation sequencing (NGS) technology, however, presents new opportunities for the field, as sequences can be elucidated in a ‘bottom-up’ manner without the need of a priori probe sequence knowledge. We used next generation sequencing (NGS) to characterize and compare the circulating transcriptomes of plasma from melanoma patients with different stage disease, along with sex-/age-matched healthy individuals, in order to identify novel biomarkers for this cancer.

We identified not only circulating miRNAs with biomarker potential, but also mRNA gene fragments and non-coding YRNAs. YRNAs (Ro-associated Y) are poorly characterized, non-coding RNAs, which were initially identified in the blood of rheumatic autoimmune disease patients [10]. They are a family based around four highly conserved sequences (*RNY1*, *RNY3*, *RNY3*, *RNY5*) involved in Ro60 inhibition, DNA replication, and quality control of non-coding RNAs [11,12,13]. Our possible biomarkers were validated in an independent cohort of 327 plasma samples from melanoma patients, collected retrospectively and prospectively. This study provides evidence that the largely unexplored circulating transcriptome could provide a valuable source of liquid biopsy biomarkers for melanoma in particular, and cancer in general.

## 2. Results

### 2.1. Sequencing the Circulating Transcriptome of Melanoma Patients

We optimized library construction by comparing several protocols, including different ribodepletion methodologies, using plasma from healthy controls in a pilot study, before settling on the protocol described in materials and methods [14]. It should be noted, however, that this protocol might result in some ligation-based bias [15]. Using this protocol, we sequenced cfRNA derived from plasma of melanoma patients, along with age-/sex-matched controls (Table 1).

**Figure 1:**
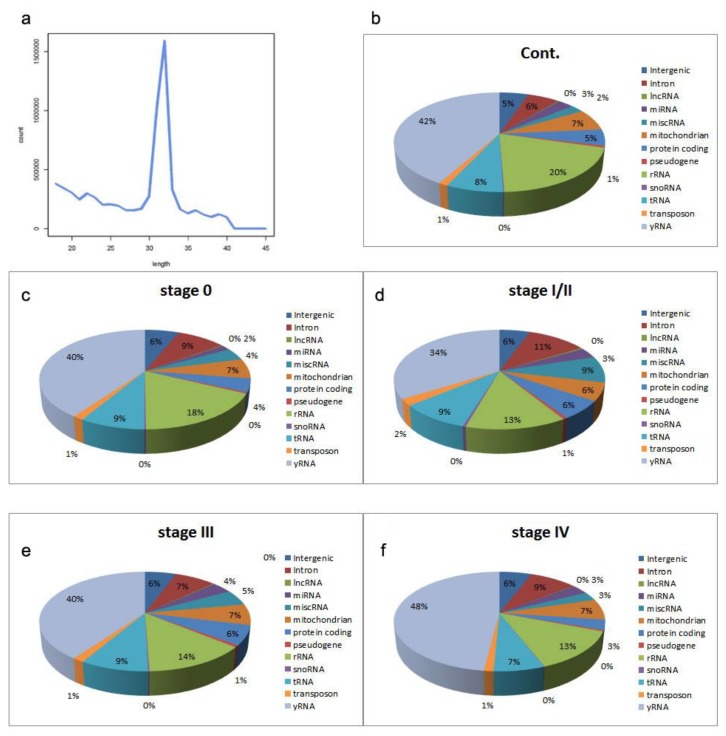
RNAseq results from circulating transcriptome. (**a**) Typical length–frequency of obtained reads in library. Proportion of reads mapping to different categories of sRNA for (**b**) control pool, (**c**) stage 0 pool, (**d**) stage I/II pool, (**e**) stage III pool, (**f**) stage IV pool.

Due to the low quantity of cfRNA in plasma, we decided to pool plasma samples according to clinical stage, as depicted in Table 1. For each pooled sample, 40–50 million reads were obtained, with an average Phred score of 37.9 (Supplementary Table S1). Between 50–55% of reads were mapped to the human genome (hg19), and approximately half of these sequences were considered small RNA (sRNA), representing sequences between 18–43 nt (Supplementary Table S1; Figure 1a). By far the largest category by frequency of reads was YRNAs, accounting for an average of 40.6% of reads (range 34–48%; Figure 1). Indeed, this category accounted for >95% of reads that composed the major peak at 32 nt seen in the size profiles of samples (typical example shown in Figure 1a).

### 2.2. miRNA Expression

We detected 442 different human miRNA sequences in the samples, which accounted for between 1.38 × 10^5^ and 3.14 × 10^5^ reads per sample. The most abundant miRNAs were members of the let-7 family, which accounted for >70% of mapped miRNAs reads, followed by miR-423 and miR-320a-3p. Eleven miRNAs were identified by ANOVA as being differentially expressed between the different disease stages and healthy controls (*p* < 0.05, >50 reads per sample; Table 2). We measured levels of the four most differentially expressed miRNAs (miR-134-5p and miR-320a-3p, miR-21-5p and miR-92b-3p) by qRT-PCR plasma samples in 28 healthy controls and 96 melanoma patients (Table 1). We were unable to detect miR-21-5p and miR-92b-3p [16].

**Figure 2:**
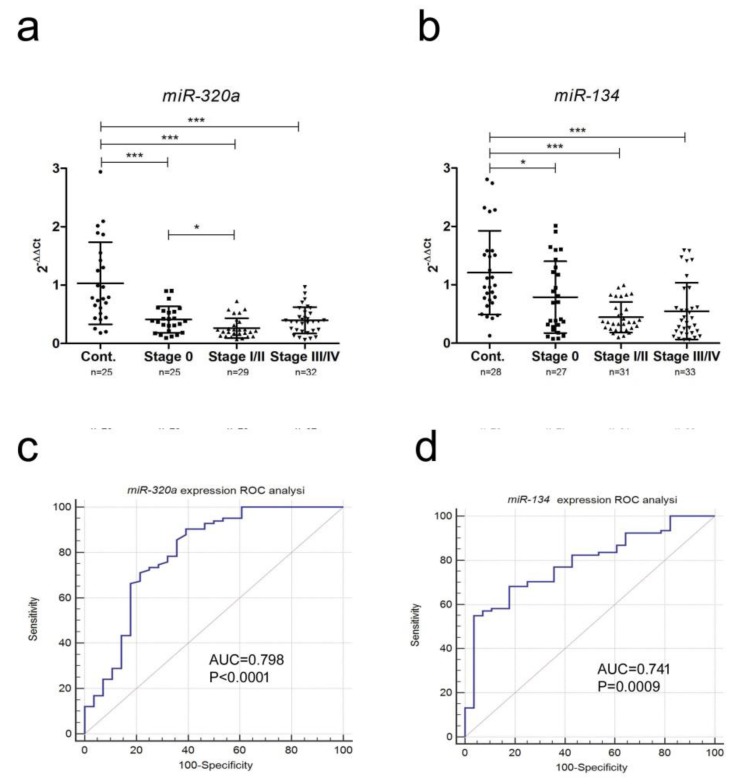
Expression levels of miRNAs measured by qRT-PCR in independent validation cohorts. (**a**,**b**) Levels of *miR-320a* and *miR-134* were measured in a cohort of 124 plasma samples (28 controls and 96 melanoma patients). Expression levels were compared using the Kruskal–Wallis multiple comparison test (both *miR-134* and *miR-320a* had *p*-values of <0.0001) and the Mann–Whitney independent *t*-test to carry out a pairwise comparison between groups (* *p* < 0.5; *** *p* < 0.001) (**c**,**d**) ROC analysis of miRNA probe expression levels as diagnostic biomarker (i.e., control vs. all melanoma patients, regardless of stage).

Consistent with the NGS data, we found that miR-134-5p and miR-320a-3p were significantly down-regulated in patients compared to controls (Figure 2a,b). Levels of these miRNAs were lower in stage 0 patients than in healthy controls, and even lower in stage I/II patients (*p* < 0.05). Receiver operating characteristic (ROC) analysis gave area under curve (AUC) values of 0.798 (miR-320a-3p) and 0.788 (miR-134-5p), with a higher sensitivity (90%) for miR-320a-3p and a higher specificity for miR-134-5p (96%) (Figure 2c,d and Table 3).

### 2.3. mRNA Fragment Expression

**Figure 3:**
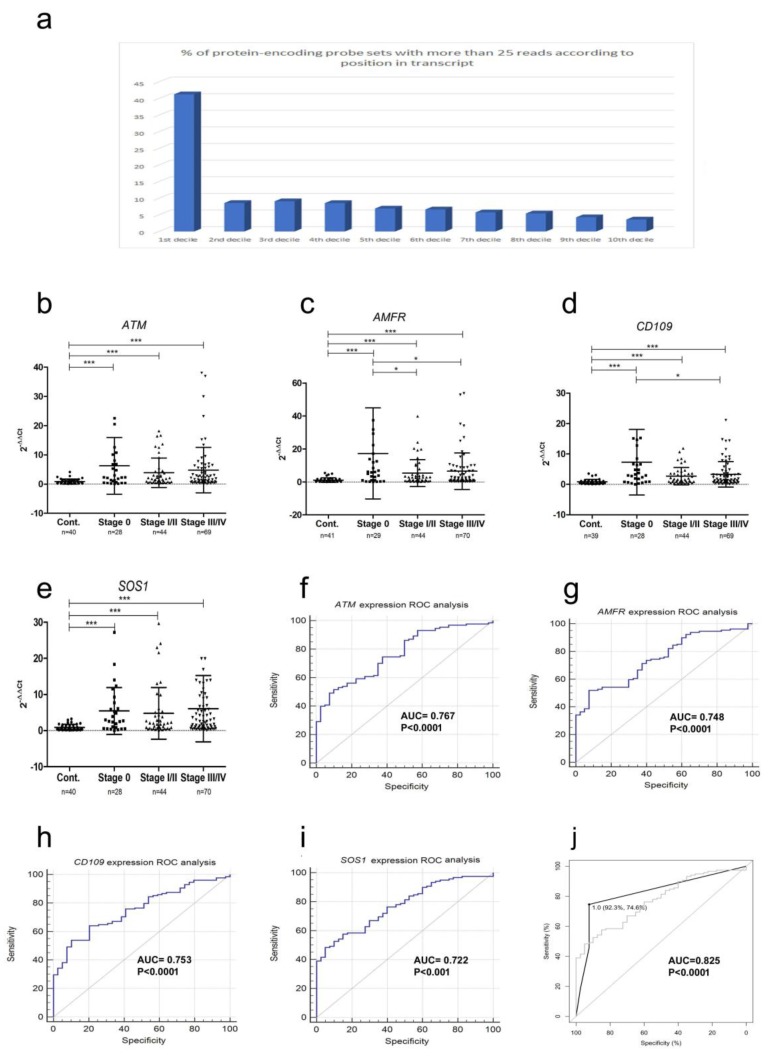
Expression levels of mRNA fragments measured by qRT-PCR in independent validation cohorts. (**a**) Proportions of probe sets containing at least 25 reads (>500 bases in length) relative to their position (5′ to 3′ direction) along the respective transcript. (**b**–**e**) Levels of *ATM*, *AMFR*, *CD109*, and *SOS1* probes were measured in a cohort of 185 plasma samples (41 controls and 144 melanoma patients). Levels are shown relative to the mean expression of the control sample cohort (i.e., 2−ΔΔ*C*t). Expression levels were compared using the Kruskal–Wallis multiple comparison test (all the mRNA fragments had *p*-values of <0.0001), and the Mann–Whitney independent *t*-test to carry out a pairwise comparison between groups (* *p* < 0.5; *** *p* < 0.001) (**f**–**i**) ROC analysis of mRNA probe expression levels as diagnostic biomarker (i.e., control vs. melanoma patient). (**j**) Panel (*ATM*, *AMFR*, and *SOS1*) performance shown with black line, and SOS1 is shown as a gray line for comparison.

Because mRNA is degraded by RNase activity in the blood into fragments with an average size of 200 bases [8], rather than mapping reads to whole genes or exons, we mapped them to short annotated probe sets (<500 bases). There were 3672 different probe sets (median length of 244 nt), with greater than 25 reads per probe set mapping to 13,641 different transcripts. For each probe set we analyzed their relative position within the respective transcripts as a percentage of the entire transcript length. By far the largest proportion (5350/13641 (39%)) of probe sets mapped to the first decile (10%) (i.e., 5′-end) of the transcript, compared to an average of 6% for the other nine deciles of the transcript length (Figure 3a).

Seventy of the probe sets were identified as being differentially expressed between samples (*p* < 0.05; Table A1). We designed custom Taqman probes to detect fragments from the five most differentially expressed probe sets (i.e., corresponding to *ATM*, *ARHGAP*, *AMFR*, *CD109*, and *SOS1* genes) (Supplementary Table S2; Figure S1). We were unable to design a specific probe for the *ARHGAP* probe set due to a high level of repetitive sequences. None of the mRNAs validated are predicted targets of miRNAs studied (i.e., *miR-134-5p* and *miR-320a*) using the predictive algorithms TargetScan and miRDB [17]. The four probe sets were measured by qRT-PCR in 47 control and 173 melanoma patient samples (Table 1).

Levels of *ATM*, *AMFR*, *CD109*, and *SOS1* were all significantly higher (*p* < 0.001) in plasma samples from either stage 0, stage I/II, or stage III/IV melanoma patients than in healthy controls (Figure 3b–e, respectively), consistent with the NGS data (Table A1). Surprisingly, levels of *AMFR* and *CD109* were higher in plasma from stage 0 patients than samples with more advanced disease. We carried out ROC analysis to determine the diagnostic ability of the mRNA fragments (Table 3; Figure 3f–i), and to discriminate between different disease stages (Table 3; Supplementary Figure S2a–d). We looked at combinations of these biomarkers using the PanelomiX ROC comparison algorithm [18]. A combination of *ATM*, *SOS1*, and *AMFR* with cut-off values of 2.13, 2.96, and 2.26 respectively, gave the best diagnostic accuracy (AUC = 0.825) (Table 3, Figure 3j).

### 2.4. YRNA Expression

**Figure 4:**
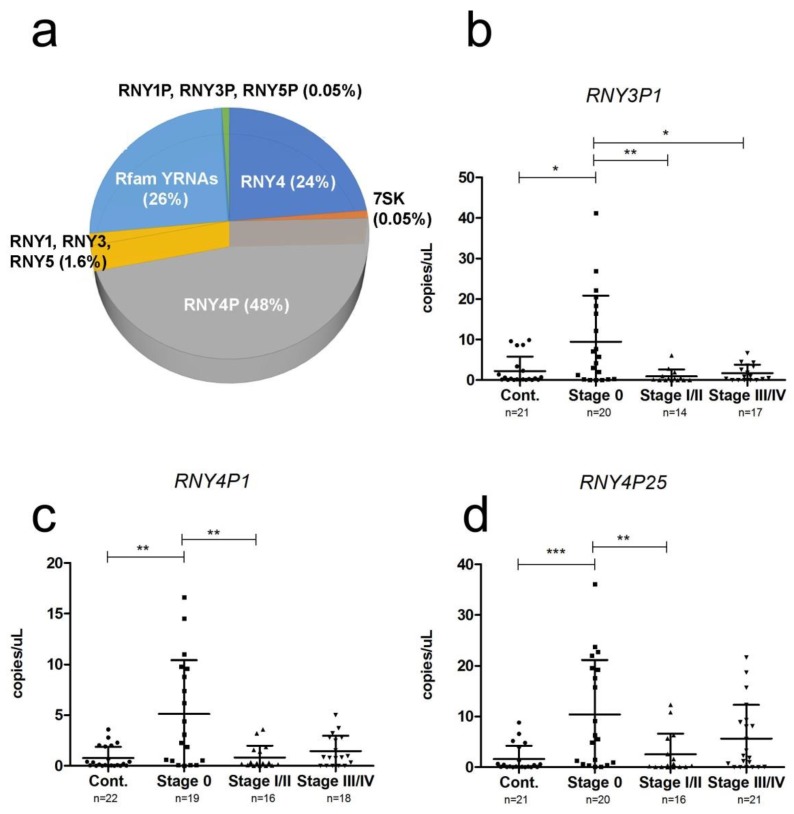
YRNA expression in plasma samples. (**a**) Mean proportions of different YRNA species in NGS cohort plasma samples. Levels of (**b**) *RNY3P1*, (**c**) *RNY4P1* and (**d**) *RNY4P25* measured by ddPCR in an independent validation cohort of 80 plasma samples (22 controls and 58 melanoma patients). Values are shown as absolute copies per µl. Expression levels were compared using the Kruskal–Wallis multiple comparison test (all the YRNA fragments had *p*-values of < 0.001), and the Mann–Whitney independent *t*-test to carry out a pairwise comparison between groups, (* *p* < 0.5; **p < 0.01*** *p* < 0.001).

As nearly half of all mapped sRNA reads were identified as YRNA sequences (Figure 1), we examined this class of non-coding RNAs further. There were 322 different YRNA and YRNA-associated sequences identified in our samples, consisting of three YRNA sequences (*RNY1*, *RNY3*, and *RNY4*) representing an average of 26.1% of reads, 30 YRNA pseudogenes representing an average of 48.4% of reads, 69 7SK sequences (average 0.05% of reads), and 194 Rfam predicted YRNA sequences representing an average of 25.5% of reads (Supplementary Table S3). The vast majority of reads were represented by *RNY4* and *RNY4P* sequences, accounting for >98% of their respective YRNA class (Figure 4a). 

We compared the expression of YRNAs between low-stage disease (i.e., stage 0 and I/II) and high-stage disease (i.e., stage III and IV), and identified five differentially expressed YRNA fragments (*p* < 0.05; Table 4). We designed custom Taqman probes to measure three of these YRNAs (*RNY3P1*, *RNY4P1*, and *RNY4P25*), selected on the basis of fold-change and read count. We measured levels of the YRNAs in a validation cohort of 80 samples (22 controls and 58 melanoma patients, Table 1). Levels of all three YRNAs were significantly higher in stage 0 samples than control samples or stage I/II samples (Figure 4b–d).

## 3. Discussion

The presence and relative stability of cfRNA in biological fluids has led to a great deal of interest in their use as ‘liquid biopsies’ for disease, in particular for cancer. However, with the exception of miRNAs, the circulating transcriptome remains largely unexplored. While NGS offers researchers the ability to elucidate the circulating transcriptome in its entirety, and therefore to identify novel biomarkers of disease, the application of RNAseq to biofluids such as plasma poses many challenges, not least the low quantity and quality of RNA present in these samples. As a consequence, studies to date have focused on the technical optimization of these techniques [19,20,21]. However, very few studies to date have sought to assess the potential usefulness of their findings through validation in independent cohorts.

In order to fully explore the complexity and biomarker potential of the melanoma circulating transcriptome, we pooled samples to maximize the starting quantity of cfRNA. As a result, we were able to obtain 40–50 million reads per pooled sample, an order of magnitude higher than comparable studies [20,22]. In contrast to exosomal cfRNA [22], we found that miRNAs only represented a minor component (<3%) of the whole plasma circulating transcriptome, levels similar to other plasma NGS studies [19,20,21]. We identified 442 different miRNAs in our samples, somewhat higher than that reported in comparable studies [23,24], probably as a result of the pooled design and the higher quantity of RNA that we used. Consistent with other studies, we found that *let-7b*, *miR-423*, and *miR-320a-3p* were the most highly expressed miRNAs in our plasma samples [20]. We identified eleven miRNAs that were differentially expressed between healthy controls and the different clinical stages of melanoma (Table 2). This included *miR-21*, which has previously been shown to be upregulated in melanoma plasma samples [25], and *miR-92b* and *miR-628*, both of which are more highly expressed in plasma from monosomy 3 uveal melanoma patients [26].

Based on our sequencing results, we measured the expression of *miR-320a-3p* and *miR-134-5p*, the two miRNAs most differentially expressed between samples, in a validation cohort of 96 melanoma patients and 28 controls. Both miRNAs were significantly down-regulated (*p* < 0.0001) in plasma from all stages of melanoma patients when compared to samples from healthy controls. *MiR-320a* has also been found to be down-regulated in melanoma tumor cells when compared to heathy skin samples [27]. Furthermore, this miRNA was shown to function as an inhibitor of cell proliferation. The down-regulation of *miR-320a* has been observed in the blood of several cancers including colorectal cancer [28], gastric cancer [29], and retinoblastoma [30]. Moreover, *miR-320a* is up-regulated in melanoma cells after treatment with bevacizumab or rapamycin + bevacizumab [31]. Furthermore, *miR-134* has been characterized as a tumor suppressor, acting to regulate proliferation, apoptosis, and invasion and migration in a wide range of cancer types, including melanoma [32,33]. ROC analysis of miRNA expression gave AUC values of 0.798 and 0.788 respectively. The *miR-320a* miRNA had a sensitivity of 90%, whereas *miR-134* had a specificity of 96%, suggesting these two miRNAs in combination could be useful biomarkers for melanoma.

Even though circulating extracellular mRNA was first detected in 1999 (in melanoma) [34], as the vast majority of circulating mRNA is degraded by blood RNase activity [35], this potential source of biomarkers has largely been overlooked, even though mRNA fragments can represent up to 75% of total cfRNA [19]. In our study, just over 5% of mapped reads corresponded to protein-encoding mRNA fragments. We detected 3672 probes (<500 bases in length) that had at least 25 mapped reads in our samples. Nearly 40% of the probes mRNA fragments mapped to the 5´-terminus (i.e., first 10%) of their respective gene transcripts, probably reflecting the 3´to 5´ cleavage activity of RNase A, the major RNase species in blood [26]. We did not notice a corresponding shift in the length profile between healthy and melanoma patient samples [36].

Pathway analysis of the genes corresponding to differentially-expressed mRNA fragments showed significant enrichment in the angiopoietin, p21-activated kinase (PAK) and Eukaryotic Initiation Factor 2 (EIF2) pathways. It has been previously reported that circulating level of Angiopoietin-2 (Ang-2) protein in melanoma patient sera closely correlates with disease progression [37]. Similarly, amplification of the PAK (p21-activated kinase) pathway is characteristic of BRAF-wild type melanoma [38], while in BRAF-mutant melanoma it is responsible for resistance to MAPK-inhibitor treatment [39]. Interestingly, both *SOS1* and *ATM1*, which were the third and fourth most differentially expressed probe sets in our analysis, form part of the angiopoietin, PAK, and EIF2 pathways. We measured levels of *SOS1*, *ATM1*, *CD109*, and *AMFR* mRNA fragments in plasma samples from 173 melanoma patients and 47 healthy controls. With the exception of *CD109*, these mRNA fragments mapped to regions corresponding to the 5′-terminus of the reference gene transcript and included the initiation codon. Consistent with the NGS data (Table A1), levels of all these mRNA fragments were up-regulated in melanoma patient samples compared to samples from healthy controls. Particularly intriguing was the up-regulation of *CD109* and *AMFR* in stage 0 samples compared to samples from more advanced stage melanoma, suggesting that these mRNA fragments could be used for early diagnosis of melanoma, although we do not have data on how many of these patients went on to develop advanced disease. (Figure 3b–e). *CD109* has been identified as an important regulator of the Epithelial–mesenchymal transition (EMT pathway), and has also been found to be down-regulated in more advanced stage hepatocellular carcinoma [40]. The product of the *AMFR* gene, gp78, also regulates EMT, and increasing levels of *AMFR* are associated with metastatic melanoma [41,42]. Intriguingly, *CD109* is a predicted target of *miR-134* by the Targetscan algorithm; we are currently carrying out experiments to confirm this. ATM1 is a serine/threonine kinase induced by DNA damage and associated with risk in many cancer types [43]. SOS1 is a guanine nucleotide exchange factor for RAS proteins frequently mutated in melanoma [44]. Interestingly, all four of these gene fragments were more highly expressed not only in advanced stage disease, but also stage 0 disease; indeed, levels of *CD109* and *AMFR* were higher in plasma from stage 0 disease than more advanced stage disease, suggesting that these biomarkers maybe non-tumoral in origin. Consistent with this hypothesis, the release of *CD109* by bone marrow mesenchymal stem cells has recently been shown to attenuate EMT in skin squamous cell carcinoma [45], and *AMFR* plays an important role in regulation of the anti-cancer immune Stimulator of interferon genes (STING) pathway [46].

To test the potential diagnostic ability of these biomarkers, we carried out ROC analysis, however the results from individual mRNA fragments were poor (AUC range 0.722 (*SOS1*) to 0.767 (*ATM1*). In contrast, a combination of *ATM1*, *SOS1*, and *AMFR* resulted in an AUC value of 0.825 with a sensitivity of 75% and specificity of 92%. Although these findings need to be confirmed independently, this combination compares very favorably with existing sera markers such as LDH and S100B with reported sensitivities/specificities of 41.6/84.2% and 36.3/96.5%, respectively [47].

By far the largest class of circulating cfRNA that we identified in the samples corresponded to YRNA sequences, accounting for close to 50% of sRNA mapped reads. Remarkably, despite the prevalence of cfYRNAs in the blood, there is very little known about this class of ncRNA. YRNAs are short 80–110 nt ncRNAs, first identified in the early 1980s as an RNA component of the soluble Ro60 ribonucleoprotein particle found in the blood of patients with autoimmune diseases [48]. The function of YRNAs is still poorly understood; they appear to be essential for DNA replication [49] and are up-regulated in cancers [50], presumably as a result of their association with apoptosis [51]. The first description of circulating cfYRNAs came in 2013 from Dhahbi et al., who observed that 33% of mapped reads from sera of healthy individuals were YRNA sequences [52]. The same group later reported that YRNA accounts for 38% of cfRNA in sera from breast cancer patients [53], and subsequently, in the sera of head and neck squamous cell carcinoma patients [54]. More recently, a study of 183 plasma samples from healthy individuals found that YRNAs accounted for 63% of cfRNA [55]. As far as we are aware, apart from a recent study that measured YRNAs in the sera of 30 renal carcinoma patients [56], this the first study to look at the biomarker potential of YRNAs in cancer patients. Interestingly, we found that levels of YRNAs were significantly higher in samples from patients with stage 0 disease, maybe pointing to increased levels of tumor-associated apoptosis [51] even despite the small tumor sizes compared to more advanced disease stages.

In summary, we have elucidated the circulating transcriptome of plasma samples from melanoma patients and found a number of novel RNA biomarker species that we validated independently using qRT-PCR and ddRT-PCR in combined retrospective and prospective cohorts, detected from only 1mL of serum. These findings have potential clinical utility as new tools for early detection of melanoma, particularly as our results suggest that these biomarkers can detect disease much earlier than current diagnostic techniques. Furthermore, as blood-based biomarkers, there is potential for screening of non-symptomatic individuals. Whilst it is clear that much further validation is required, this study provides strong evidence that the circulating transcriptome holds much promise as a source of liquid biopsies for melanoma that surely merits further exploration.

## 4. Materials and Methods

### 4.1. Patient Cohorts

Patient plasma samples were collected both retrospectively (*n* = 119) and prospectively (*n* = 289). Retrospective samples were obtained from the John Radcliffe Hospital, Oxford (Oxford cohort; *n* = 30), and the AVAST-M multi-center phase 3 clinical trial (AVAST-M cohort; *n* = 89) [57]. Samples collected prospectively came either from the Hospital 12 de Octubre in Madrid (Madrid cohort; *n* = 67) or the Onkologikoa Cancer Hospital and Donostia University Hospital of San Sebastián (San Sebastián cohort; *n* = 102): a total of 327 melanoma patients (Table 5). The clinical details of the individual patients included in these cohorts are given in Supplementary Tables S4–S7. Samples were collected at the time of diagnosis and prior to any treatment. Unfortunately, no information was available regarding the sequence of *CDKN2A*. Plasma samples from age/sex matched healthy controls (*n* = 99) were obtained from the Basque Biobank for Research O+EHUN. Plasma preparation was carried out within 1 h of phlebotomy, with blood collected in EDTA-coated tubes followed by centrifugation for 1000× *g* for 15 min at 4 °C.

With the exception of the 37 prospectively collected samples used for NGS (Table 1), only a limited volume (1–2 mL) of plasma was available for validation studies. Therefore, we divided samples into three separate validation cohorts; a miRNA cohort of 96 melanoma patients and 28 controls; an mRNA cohort of 173 melanoma patients and 47 controls; and a YRNA cohort of 58 melanoma patients and 22 controls. The clinical details of the patients used are summarized in Table 1, and details of individual cohorts are provided in Table 4 and Supplementary Tables S4–S7. Written informed consent was obtained from patients for the inclusion of their samples in this study, and samples were collected in accordance with the Declaration of Helsinki and approved by local ethics committees (CEIC-Euskadi approval number PI2015024).

For the plasma samples used for NGS (5–8 mL), cfRNA was purified using the plasma RNA purification kit from Norgen Biotek (Ontario, Canada), and for validation studies (1 mL samples) the miRCURY™ RNA Isolation Kit Biofluids from Exiqon (Vedbaek, Denmark) was used.

### 4.2. Library Construction and Next-Generation Sequencing

The samples used for NGS were pooled according to disease stage, as shown in Table 1. Ribosomal RNA (rRNA) was removed from total cfRNA using the Ribozero Magnetic Human/Mouse/Rat kit (Epicentre (Maidon, WI, USA), #MRZH116), according to the low input protocol recommended by the manufacturer. Phosphatase and T4 polynucleotide kinase (PNK) treatments were carried out on the ribo-depleted RNA, and Illumina small RNA adapters ligated. Libraries were amplified using 15 cycles of PCR of barcoded primers [58]. Sequencing was performed on an Illumina HiSeq 2500 as 50 PE in rapid mode.

### 4.3. Bioinformatic Analysis

Sequencing reads were quality filtered using the fastx_artifacts_filter tool, and ligation adapters were removed using the AdRec.jar program (seqBuster suite of programs (omicX (Le-Petit-Quevilly, Le Petit-Quevilly, France))). Reads were mapped to the GRCh37 build of the human genome using the Bowtie 2.0 algorithm. A custom annotated probe set was built by combining probes from GENCODE version 8 [59], supplemented with rRNA and repeat annotations from RepeatMasker GRCh37, and snoRNA annotations from the UCSC table browser [60]. Expression of miRNA was calculated using the miraligner algorithm from the seqBuster suite, and YRNA expression was calculated using the HTseq-count algorithm. Differential expression analysis was carried out using the DESeq bioconductor package [61].

### 4.4. qRT-PCR (mRNA and miRNA) and ddPCR(yRNAs)

mRNA was reverse transcribed (RT) using random primers with the High Capacity cDNA Reverse Transcription Kit from Applied Biosystems following the manufacturers’ protocol with DNase treatment. Due to the difficulty in quantifying cfRNA reliably, we used fixed volumes in reactions [4,62]. For gene fragments, we designed custom Taqman probes using the Custom TaqMan^®^ Assay Design Tool from Applied Biosystems (Foster City, CA, USA), using the sequences corresponding to the respective probe sets (Supplementary Table S2 and Figure S1). The reference gene used for mRNA analysis was 18S rRNA, as previously described [63].

For miRNA detection, we used the Megaplex RT Primers Human Pool A v2.1 for RT, and specific Taqman probes as described in the text. As the problems of defining suitable reference genes for miRNA detection in plasma are well documented [4], we measured levels of three previously described reference miRNAs (*miR-24*, *miR-16* and *miR-191*) [64,65], in a cohort of 63 samples (control/stage 0 (*n* = 22), stage I/II (*n* = 26), stage III/IV (*n* = 15)). Using the NormFinder algorithm we identified miR-24 and miR-191 as the most stable combination of reference genes in our samples (Supplementary Figure S3).

Custom primers and Taqman probes for Y3P1, Y4P1 and Y4P25 were designed using the Custom Taqman^®^ Small RNA Assay Design Tool from Applied Biosystems. We performed ddPCR using QX200TM Droplet DigitalTM PCR system (Bio Rad, Hercules CA, USA), following the manufacturers’ protocol. Data analysis was performed by QuantaSoft analysis software from Bio-Rad. Expression levels were compared using the Kruskal–Wallis multiple comparison test, and the Mann–Whitney independent t-test to carry out a pairwise comparison between individual groups (Graphpad Prism v. 5.0, La Jolla, CA, USA). ROC analysis and comparisons were carried out using the method of DeLong et al., as implemented in MedCalc v. 14.8 software [66].

## 5. Conclusions

We have carried out a comprehensive, non-biased elucidation of the circulating transcriptome of melanoma patients and identified a number of promising candidate biomarker RNA species, not only miRNAs. These candidates were validated in independent cohorts by ourselves, however it is clear that further studies should be carried out by independent research groups in order to strengthen our findings and facilitate the translation of this knowledge into the clinic.

What is obvious is that many virtually unexplored classes of the circulating transcriptome are yet to be fully assessed for their ability to serve as useful cancer biomarkers. As a consequence, while the discovery of circulating miRNAs represented an important event in the history of the liquid biopsy field, it is clear that there is much that we have still to explore.

## Figures and Tables

**Table 1:** Summary of clinical details of patient cohorts used in study. NK: not known.

	Stage	*n*	Age (Median)	Sex (M/F/(NK))
	Control	8	58	4/4
	Pool 0	5	76	3/2
**NGS cohort**	Pool I/II	8	63	4/4
	Pool III	8	68	3/5
	Pool IV	8	59	3/5
	Control	47	54	18/23
**mRNA**	Stage 0	34	64	11/17/(1)
**validation cohort**	Stage I/II	52	58	19/25
	Stage III/IV	87	57.5	32/37/(2)
	Control	28	58	12/14/(2)
**miRNA**	Stage 0	29	51	6/19/(4)
**validation cohort**	Stage I/II	33	60	10/14/(9)
	Stage III/IV	34	55	11/15/(8)
	Control	22	57.5	11/11/
**YRNA**	Stage 0	20	51	11/5/(4)
**validation cohort**	Stage I/II	17	48	9/8
	Stage III/IV	21	61	8/13
**Total**	**-**	**426**	**58**	**175/221/(30)**

**Table 2:** Differentially expressed microRNAs (miRNAs) (*p* < 0.05). Counts were normalized per million reads. miRNAs validated are depicted in bold.

miRNA	Cont.	Stage 0	Stage I/II	Stage III	Stage IV	*p*-Value
*miR-134-5p*	7474	7290	2293	4200	4876	0.0158
*miR-320a-3p*	117,411	76,952	56,705	86,933	74,154	0.0181
*miR-21-5p*	356	405	368	782	469	0.0183
*miR-92b-3p*	14	14	20	25	51	0.0232
*miR-98-5p*	9850	12,999	9124	14,243	13,627	0.0232
*miR-16-3p*	21	29	49	39	53	0.0277
*Let-7b*	385	507	326	267	396	0.0286
*miR-1827*	58	28	7	7	11	0.0375
*miR-1180*	107	145	49	45	42	0.0392
*miR-628*	499	667	375	502	407	0.0496
*miR-486*	4581.23	6014.45	1501.33	2704.89	1879.67	0.0497

**Table 3:** ROC analysis values of expression levels of differentially expressed miRNAs and gene fragments. * AUC values of different stage vs. control sample. Panel was a combination of *ATM*, *AMFR*, and *SOS1*.

Probe	AUC	Sensitivity	Specificity	95% CI	0 *	I/II *	III/IV *
*miR-320a-3p*	0.798	90%	61%	0.712–0.869	0.751	0.870	0.828
*miR-134-5p*	0.788	55%	96%	0.704–0.858	0.680	0.868	0.811
*ATM*	0.767	61%	72%	0.697–0.829	0.769	0.734	0.715
*AMFR*	0.748	52%	92%	0.676–0.812	0.822	0.709	0.641
*CD109*	0.753	54%	90%	0.680–0.816	0.816	0.706	0.702
*SOS1*	0.772	48%	95%	0.699–0.835	0.796	0.694	0.693
Panel	0.825	75%	92%	-	-	-	-

**Table 4:** Differentially expressed YRNAs (*p* < 0.05). Counts were normalized per million reads. YRNAs validated are depicted in bold.

	Stage 0/I/II	Stage III/IV	Fold Change	*p*-Value
*RNY4P18*	85	193	2.2	0.00033
*RNY3P1*	86	210	2.4	0.00063
*RNY4P6*	12,442	24,473	1.9	0.00072
*RNY4P1*	68	146	2.1	0.00090
*RNY4P25*	180	317	1.7	0.0032

**Table 5:** Details of origin of patient cohorts used in study.

	Cohort	*n* Samples
mRNA	Oxford	30
	AVAST-M	83
	Madrid	51
	San Sebastián	67
miRNA	AVAST-M	6
	Madrid	35
	San Sebastián	83
yRNA	Madrid	51
	San Sebastián	67

## Supplementary Materials

The following are available online at http://www.mdpi.com/2072-6694/11/1/70/s1, Figure S1: Mapping positions (GRCh37) of selected gene fragment probe sets used to design Taqman probes. Figure S2: Variation of Ct values in levels of miRNA reference genes in selected cohort measured by qRT-PCR. Figure S3: ROC analysis of mRNA expression in different melanoma stage cohorts. Table S1: Summary of NGS results from pooled RNA samples. Table S2: Characteristics of selected differentially protein-encoding gene fragment probe sets. Table S3: YRNA reads from NGS. Table S4: Clinical details of patients used for NGS cohort. Table S5: Clinical details of patients used in mRNA validation cohort. Table S6: Clinical details of patients used in miRNA validation cohort. Table S7: Clinical details of patients used in yRNA validation cohort.

## Appendix A

**Table A1:** Differentially expressed protein-encoding probe sets (*p* < 0.05). Gene names and coordinates are given. Counts were normalized per million reads. mRNAs validated are depicted in bold.

Gene	Chromosome Position	Length	*p*-Value	Cont.	St. 0	St. I/II	St. III	St. IV
*CD109*	6:74446106–74446231	125	0.00103	39	102	126	207	318
*ARHGAP*	4:148744047–148744108	61	0.0018	45	9	11	10	8
*SOS1*	2:39237725–39237844	119	0.00196	23	16	130	121	139
*ATM*	11:108099905–108100050	145	0.00316	5	20	8	7	105
*AMFR*	16:56396751–56396855	104	0.00329	11	32	59	59	121
*KCNIP3*	2:95976103–95976544	441	0.00362	1	14	22	16	28
*POLE*	12:133248801–133248908	107	0.00456	0	36	11	12	72
*KLHL7*	7:23213634–23214040	406	0.00479	0	6	10	8	40
*ZMYM4*	1:35824626–35825047	421	0.00547	54	23	25	27	22
*GTDC1*	2:144966170–144966371	211	0.00632	58	15	16	21	14
*RFWD2*	1:176132951–176133027	76	0.00651	27	11	10	13	12
*ARHGAP10*	4:148778704–148779039	335	0.00672	31	10	13	11	13
*RHBDD1*	2:227778924–227779353	429	0.00774	71	63	39	33	28
*HNRNPM*	19:8527413–8527473	60	0.00798	42	15	20	17	19
*DYNC2H1*	11:102988360–102988592	232	0.00835	0	15	17	15	60
*ITIH5*	10:7613668–7614061	393	0.00885	0	11	12	6	32
*TNFA1P2*	14:103592664–103593029	365	0.00974	29	28	84	76	60
*SLC40A1*	2:190430080–190430325	245	0.00996	21	20	31	28	34
*SETDB1*	1:150902443–150902932	489	0.0106	1	20	23	17	60
*IGF2BP3*	7: 23385559–23385780	221	0.0118	27	27	11	14	15
*DENND3*	8:142152302–142153707	205	0.0118	0	13	32	4	3
*GAK*	4:866054–866461	407	0.014	0	11	16	10	40
*ARHGEF12*	11: 120278447–120282546	99	0.015	72	78	52	39	44
*HINT3*	6: 126298790–126301387	497	0.0157	76	66	8	19	11
*HTR1E*	6: 87647124–87647541	417	0.0158	41	95	106	75	92
*UXS1*	2:106780123–106780166	43	0.0171	60	5	5	6	2
*MPP2*	17: 41983448–41983519	71	0.0173	11	9	22	29	18
*NAIP*	5: 70308275–70308745	470	0.0183	0	10	41	5	2
*SNCA*	4:90757894–90758379	485	0.0185	73	44	49	49	41
*TRAPPC9*	8:140922366–140922544	178	0.0185	28	68	76	53	59
*PISD*	22:32034352–32034488	136	0.0189	225	533	600	413	557
*SERPINB1*	6: 2836090–2836257	233	0.0193	29	14	10	14	13
*GRN*	17:42429383–42429616	167	0.0193	0	7	25	2	2
*EIF4G3*	1: 21231376–21231464	88	0.0195	49	45	38	33	33
*ILK*	11: 6624961–6625456	495	0.0196	28	49	46	59	47
*C14ORF38*	14:60031765–60031993	240	0.0197	26	7	11	9	7
*PSAP*	10:73578788–73579028	228	0.0197	1	13	27	44	13
*TMEM104*	17: 72835466–72835918	452	0.0212	46	35	11	19	16
*TAOK1*	17:27849298–27849537	239	0.0217	42	9	12	6	11
*SIAE*	11: 124506788–124507098	310	0.022	26	22	6	12	7
*CDK5RAP2*	9: 123165584–123165940	356	0.0229	4347	9583	12031	8114	9164
*TPT1*	13: 45911208–45911614	406	0.0239	55	31	38	34	30
*SSH3*	11:67070919–67071162	243	0.024	236	73	71	46	40
*SSB*	2:170667368–170667554	186	0.0247	0	8	28	7	1
*ZNF430*	19:21216892–21216990	98	0.025	48	11	17	12	8
*HBA2*	16:222846–223262	494	0.0266	266	141	135	177	134
*ZFPM2*	8:106456508–106456609	101	0.0304	70	41	43	49	49
*RBM5*	3:50145665–50145738	73	0.0308	60	19	16	28	16
*ITGB1*	10:33218750–33218972	222	0.0312	80	37	29	45	32
*ZCCHC17*	1: 31821676–31821821	145	0.0329	15	17	20	23	26
*PRKG2*	4: 82136085–82136218	133	0.0332	40	49	20	29	26
*IFI6*	1: 27992572–27992986	414	0.0341	0	0	46	1	2
*MX2*	21: 42774561–42776800	458	0.0363	0	0	31	1	1
*PSMD2*	3:184020467–184020611	144	0.0366	0	2	12	28	1
*HERC6*	4:89363186–89364063	477	0.0376	0	3	54	4	1
*PPBP*	4:74853673–74853914	241	0.0387	685	259	301	403	260
*PKN2*	1:89206671–89206971	300	0.0389	66	27	27	22	15
*TNIP1*	5: 150415143–150415268	125	0.039	7	5	36	16	14
*ARGLU1*	13: 107209096–107210043	347	0.0396	0	11	42	5	6
*LRRN2*	1:204654448–204654861	413	0.0399	13	43	29	25	32
*ACACB*	12:109625804–109625967	163	0.0401	19	46	43	30	40
*EIF5B*	2: 99980108–99980325	217	0.0415	39	14	22	18	15
*FRA10AC1*	10:95441237–95441355	118	0.0419	33	6	6	5	2
*RASA1*	5:86627165–86627317	152	0.0437	35	10	15	17	16
*CHD9*	16:53301839–53302038	199	0.0441	41	10	11	20	13
*TPM1*	15:63351762–63351879	117	0.0443	38	12	18	19	12
*ACVR2*	2:148657313–148657467	154	0.0462	26	21	16	10	14
*MCTP1*	5:94224580–94224677	250	0.0475	6	12	20	19	25
*HOXC6*	12:54404873–54407570	97	0.0481	26	62	71	45	49
*ATF4*	22:39916183–39917676	267	0.0487	26	28	18	18	18
