# Supplementary material for: The Circulating Transcriptome as a Source of Biomarkers for Melanoma
Source: Cancers (Basel). 2019 Jan 10;11(1):70. doi: 10.3390/cancers11010070 (PMC6356785; doi:10.3390/cancers11010070)
Supplement: Supplementary file 1 [file cancers-11-00070-s001.pdf]

cancers

MDPI

Supplemental Materials

The Circulating Transcriptome as a Source of Biomarkers for Melanoma

Carla Solé, Daniela Tramonti, Maike Schramm, Ibai Goicoechea, María Armesto, Luiza I. Hernandez, Lorea Manterola, Marta Fernandez-Mercado, Karmele Mujika, Anna Tuneu, Ane Jaka, Maitena Tellaetxe, Marc R. Friedländer, Xavier Estivill, Paolo Piazza, Pablo L. Ortiz-Romero, Mark R. Middleton [2] and Charles H. Lawrie

(A)

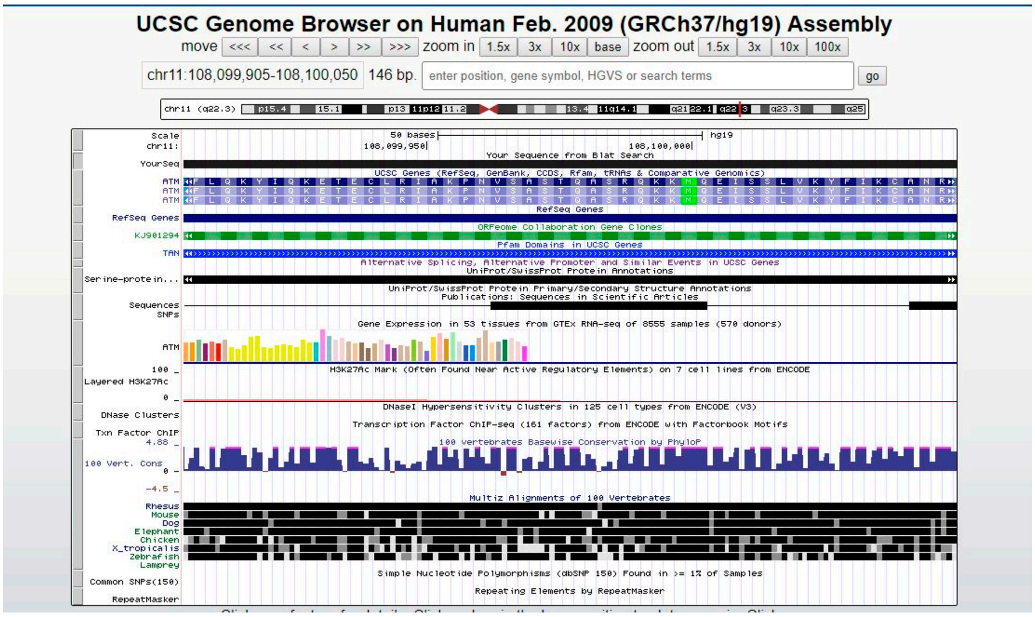

(B)

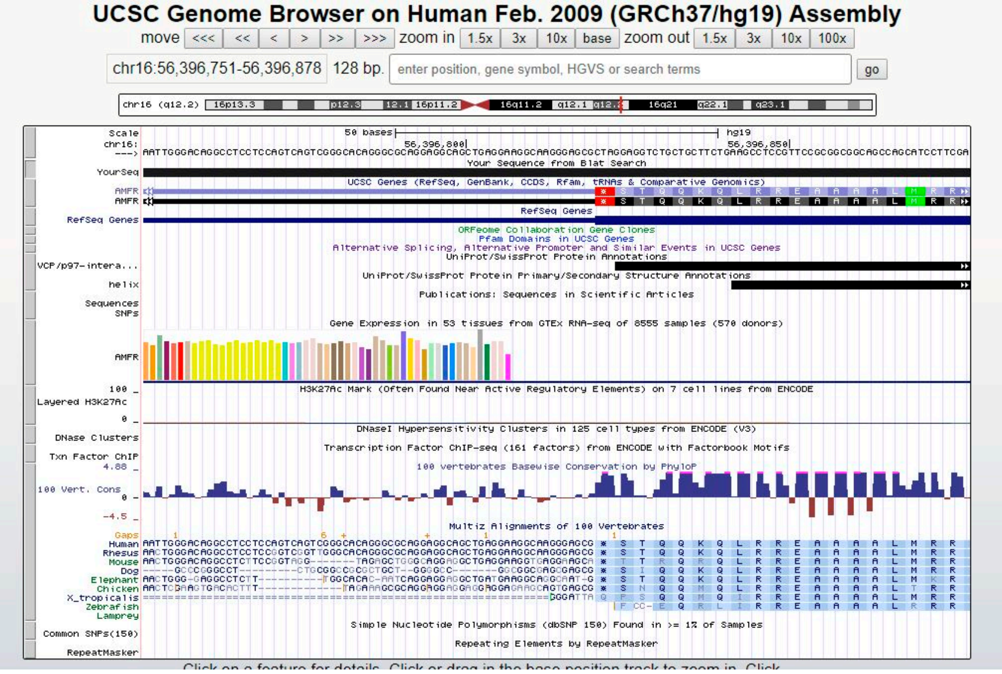

(C)

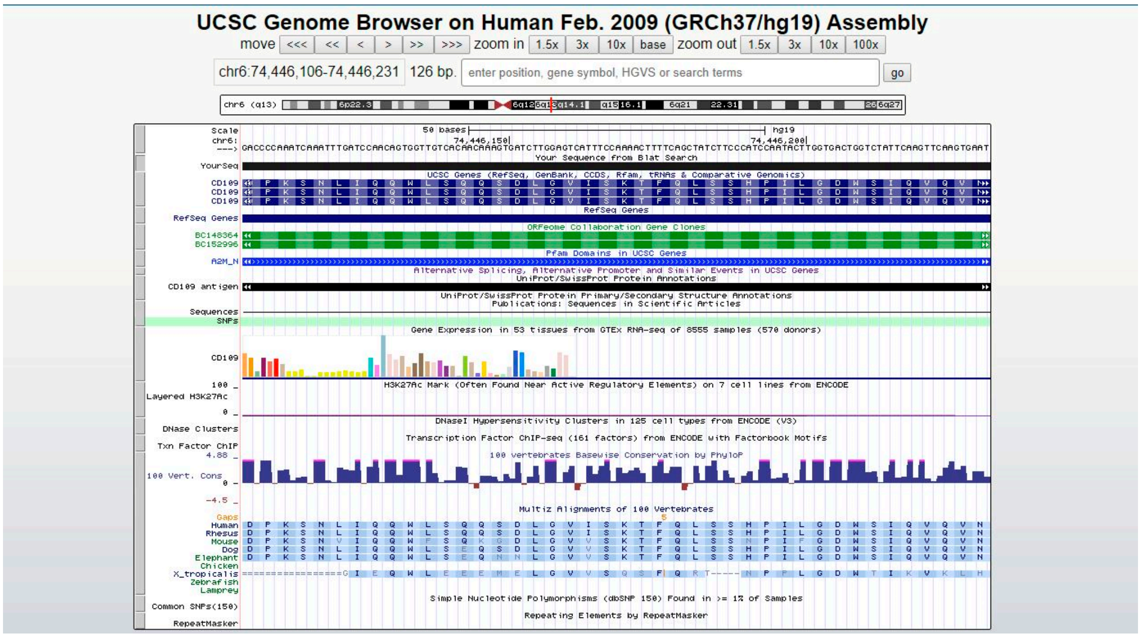

(D)

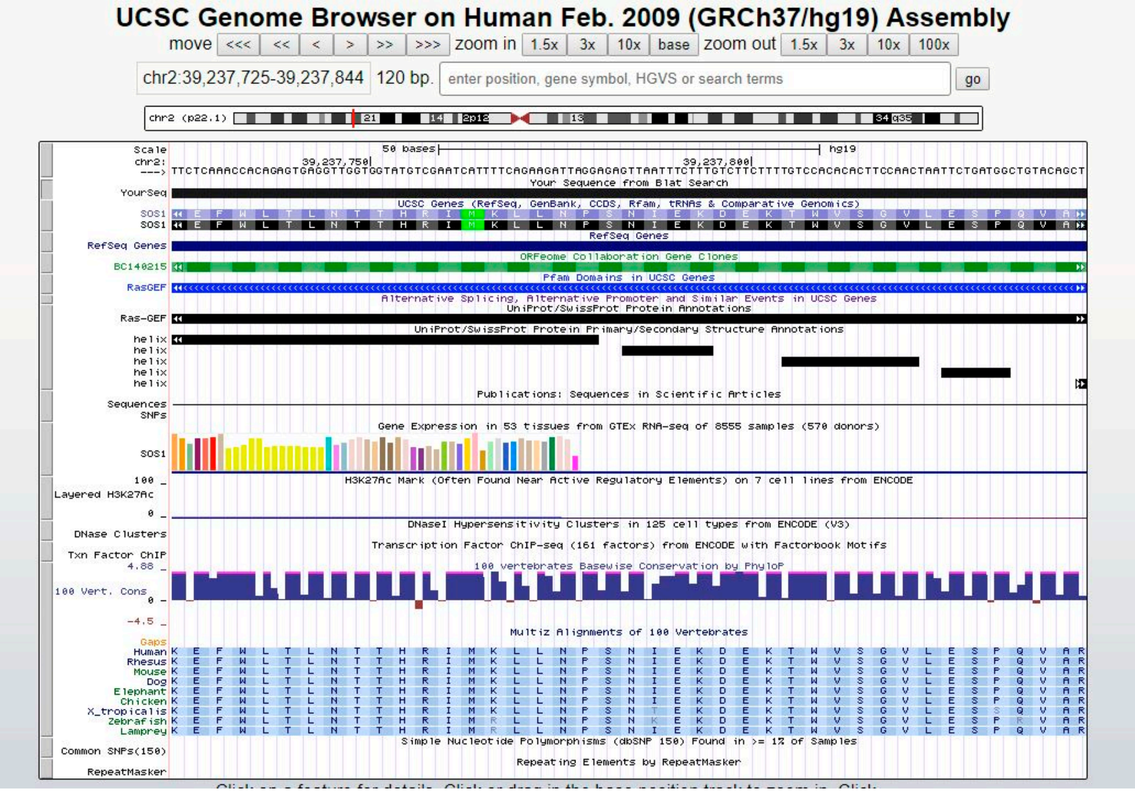

Figure S1. Mapping positions (GRCh37) of selected gene fragment probe sets used to design Taqman probes. (A) ATM (aa63-109) probe sequence, (B) AMFR (5ÚTR+aa1-10) probe sequence; (C) CD109 (aa170-211) probe sequence, (D) CD109 (aa798-836) probe sequence.

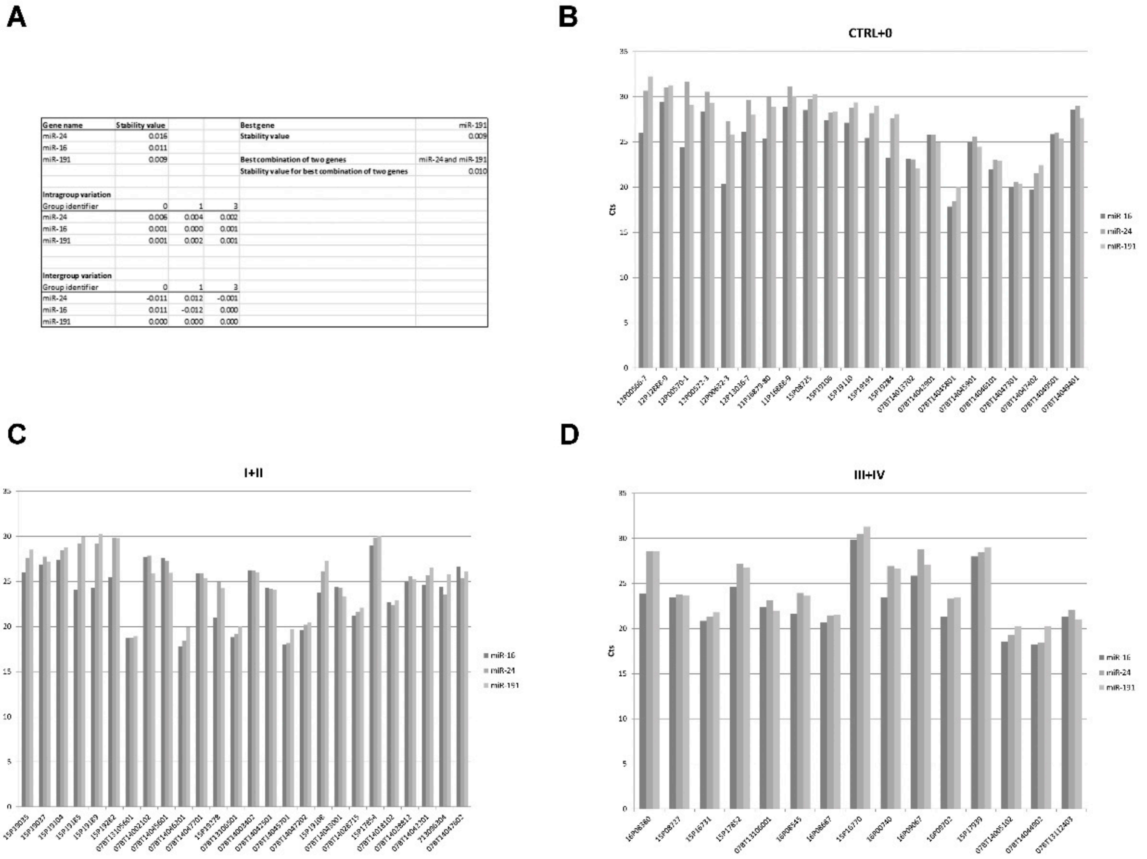

Gene name	Stability value			Best gene	miR-191
miR-24	0.016			Stability value	0.009
miR-16	0.011				
miR-191	0.009			Best combination of two genes	miR-24 and miR-191
				Stability value for best combination of two genes	0.010
Intragroup variation					
Group identifier	0	1	3		
miR-24	0.006	0.004	0.002		
miR-16	0.001	0.000	0.001		
miR-191	0.001	0.002	0.001		
Intergroup variation					
Group identifier	0	1	3		
miR-24	-0.011	0.012	-0.001		
miR-16	0.011	-0.012	0.000		
miR-191	0.000	0.000	0.000		

Figure S2. Variation of Ct values in levels of miRNA reference genes in selected cohort measured by qRT-PCR. (**A**) NormFinder analysis of levels of *miR-16*, *miR-24* and *miR-191*. (**B**) Levels of miRNAs in healthy and stage 0 patient cohort, (**C**) in stage I/II cohort and stage III/IV cohort.

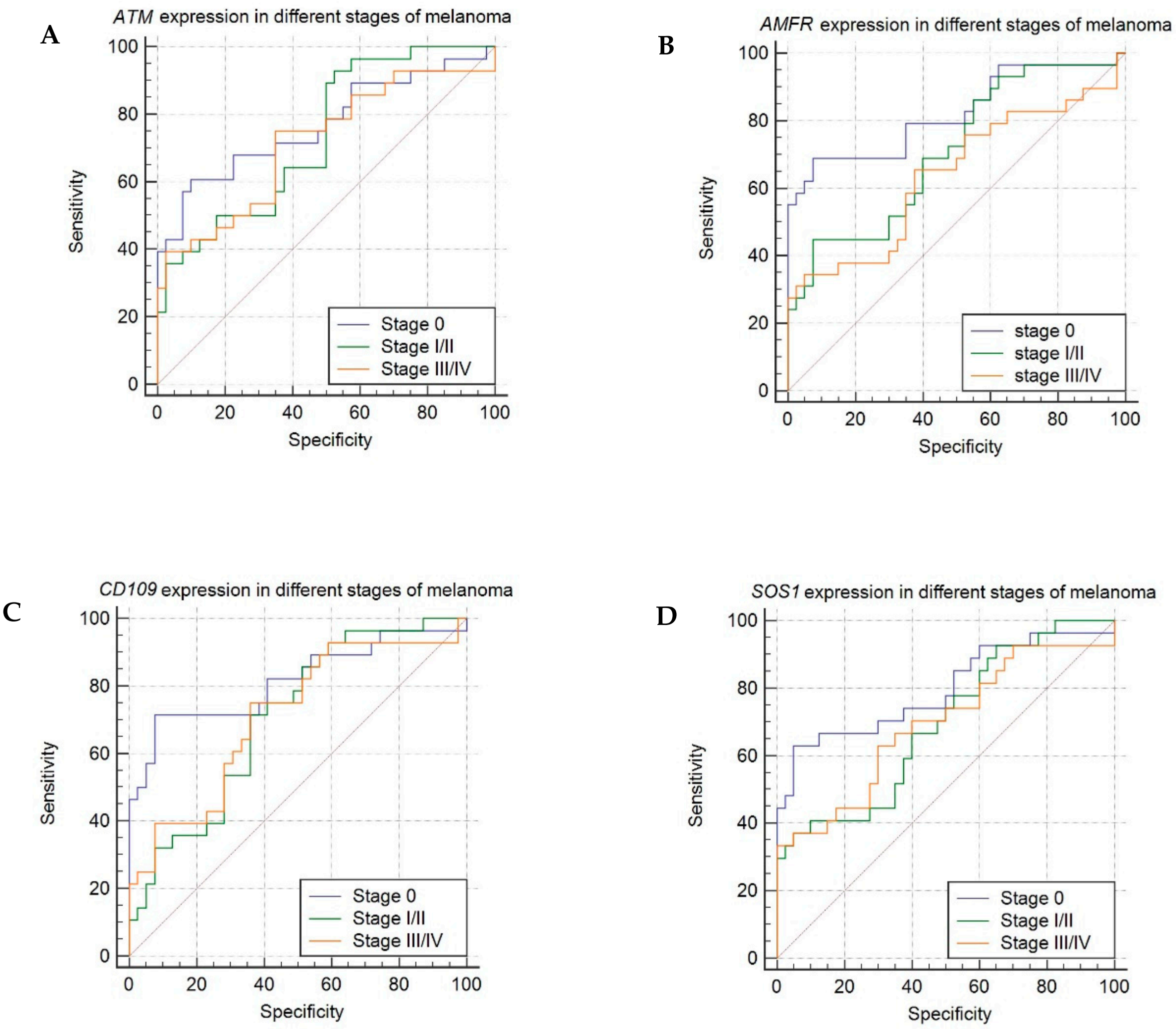

Figure S3. ROC analysis of mRNA expression in different melanoma stage cohorts. (**A**) *ATM*, (**B**) *AMFR*, (**C**) *CD109*, (**D**) *SOS1* expression.

Table S1. Clinical details of patients used for NGS cohort. NK—not known.

ID	Gender	Age	Stage	TMN
pool-0#1	Female	79	0	Melanoma in situ
pool-0#2	Female	80	0	Melanoma in situ
pool-0#3	Male	65	0	Melanoma in situ
pool-0#4	Female	58	0	Lentigo maligna in situ
pool-0#5	Male	76	0	Lentigo maligna in situ
pool-I/II#1	Female	62	IA	T1aN0M0
pool-I/II#2	Male	60	IA	T1aN0M0
pool-I/II#3	Male	82	IA	T1aN0M0
pool-I/II#4	Male	35	IA	T1aN0M0
pool-I/II#5	Female	61	IA	T1aN0M0
pool-I/II#6	Female	81	IA	T1aN0M0
pool-I/II#7	Male	84	IIIB	T4bN0M0
pool-I/II#8	Female	64	IA	T1aN0M0
pool-III#1	Female	83	IIIB	T2bN2cM0
pool-III#2	Female	67	IIIC	TxN3M0
pool-III#3	Female	48	IIIB	T2xN2cM0
pool-III#4	Male	55	IIIC	T2aN3M0
pool-III#5	Male	80	IIIB	T2aN2cM0
pool-III#6	Female	68	IIIC	T4aN0M0
pool-III#7	Female	89	IIIA	T3aN2cM0
pool-III#8	Male	61	IIIA	T3aN2cM0
pool-IV#1	Female	54	IV	T3bN0M1
pool-IV#2	Female	79	IV	T4bN1M1
pool-IV#3	Male	73	IV	T3aN3M1
pool-IV#4	Female	53	IV	T4bN1bM1
pool-IV#5	Male	58	IV	T4bN3M1
pool-IV#6	Male	51	IV	T3bN3M1
pool-IV#7	Female	60	IV	TxN3m1
pool-IV#8	Female	82	IV	TxNxM1
pool-cont#1	Female	60	-	-
pool-cont#2	Female	65	-	-
pool-cont#3	Male	57	-	-
pool-cont#4	Male	58	-	-
pool-cont#5	Male	53	-	-
pool-cont#6	Male	58	-	-
pool-cont#7	Female	62	-	-
pool-cont#8	Female	58	-	-

Table S2. Clinical details of patients used in mRNA validation cohort. NK—not known.

ID	Origin	Gender	Age	Stage	TNM
Cont#1	Biobanco	Female	64	Control	-
Cont#2	Biobanco	Female	70	Control	-
Cont#3	Biobanco	Female	67	Control	-
Cont#4	Biobanco	Female	56	Control	-
Cont#5	Biobanco	Female	68	Control	-

Cont#6	Biobanco	Male	62	Control	-
Cont#7	Biobanco	Male	57	Control	-
Cont#8	Biobanco	Male	60	Control	-
Cont#9	Biobanco	Male	63	Control	-
Cont#10	Biobanco	Male	67	Control	-
Cont#11	Biobanco	Female	59	Control	-
Cont#12	Biobanco	Female	53	Control	-
Cont#13	Biobanco	Female	55	Control	-
Cont#14	Biobanco	Female	64	Control	-
Cont#15	Biobanco	Male	54	Control	-
Cont#16	Biobanco	Male	55	Control	-
Cont#17	Biobanco	Male	54	Control	-
Cont#18	Biobanco	Male	53	Control	-
Cont#19	Biobanco	Female	29	Control	-
Cont#20	Biobanco	Female	24	Control	-
Cont#21	Biobanco	Female	26	Control	-
Cont#22	Biobanco	Male	22	Control	-
Cont#23	Biobanco	Male	28	Control	-
Cont#24	Biobanco	Female	43	Control	-
Cont#25	Biobanco	Female	31	Control	-
Cont#26	Biobanco	Female	33	Control	-
Cont#27	Biobanco	Female	34	Control	-
Cont#28	Biobanco	Female	50	Control	-
Cont#29	Biobanco	Male	42	Control	-
Cont#30	Biobanco	Male	30	Control	-
Cont#31	Biobanco	Female	45	Control	-
Cont#32	Biobanco	Male	35	Control	-
Cont#33	Biobanco	Male	50	Control	-
Cont#34	Biobanco	Female	31	Control	-
Cont#35	Biobanco	Male	NK	Control	-
Cont#36	Biobanco	Female	NK	Control	-
Cont#37	Biobanco	Male	59	Control	-
Cont#38	Biobanco	Female	54	Control	-
Cont#39	Biobanco	Female	60	Control	-
Cont#40	Biobanco	Male	53	Control	-
Cont#41	Biobanco	Female	62	Control	-
mRNA#1	Madrid	Male	77	0	-
mRNA#2	Madrid	Male	46	0	TisN0M0
mRNA#3	Madrid	Female	57	0	TisN0M0
mRNA#4	Madrid	Female	68	0	TisN0M0
mRNA#5	Madrid	Male	45	0	TisN0M0
mRNA#6	Madrid	Female	85	0	TisN0M0
mRNA#7	Madrid	Female	78	0	TisN0M0
mRNA#8	Madrid	Male	56	0	TisN0M0
mRNA#9	Madrid	Male	84	0	TisN0M0
mRNA#10	Madrid	Male	40	0	Melanoma in situ
mRNA#11	Madrid	Male	66	0	Léntigo maligno. In situ

mRNA#12	Madrid	Male	52	0	Léntigo maligno. In situ
mRNA#13	Madrid	Female	84	0	Léntigo maligno. In situ
mRNA#14	Madrid	Male	43	0	Léntigo maligno. In situ
mRNA#15	Madrid	Female	33	0	TisN0M0
mRNA#16	San Sebastián	Male	64	0	pT1sN0
mRNA#17	San Sebastián	NK	67	0	pT1sN0M0
mRNA#18	San Sebastián	Female	68	0	pT1sN0
mRNA#19	San Sebastián	Female	73	0	pT1sN0M0
mRNA#20	San Sebastián	Female	60	0	pT1sN0M0
mRNA#21	San Sebastián	Female	70	0	pT1sN0M0
mRNA#22	San Sebastián	Male	57	0	pT1sN0M0
mRNA#23	San Sebastián	Female	59	0	pT1sN0M0
mRNA#24	San Sebastián	Female	71	0	pT1sN0M0
mRNA#25	San Sebastián	Female	46	0	pT1sN0M0
mRNA#26	San Sebastián	Female	64	0	pT1sN0M0
mRNA#27	San Sebastián	Female	76	0	TisN0M0
mRNA#28	San Sebastián	Female	75	0	TisN0M0
mRNA#29	San Sebastián	Female	40	0	TisN0M0
mRNA#30	Madrid	Male	47	IA	T1aN0M0
mRNA#31	Madrid	Female	72	IA	T1aN0M0
mRNA#32	Madrid	Female	81	IA	T1aN0M0
mRNA#33	Madrid	Female	43	IA	T1aN0M0
mRNA#34	Madrid	Female	37	IA	T1aN0M0
mRNA#35	Madrid	Male	42	IA	T1aN0M0
mRNA#36	Madrid	Male	57	IA	T1aN0M0
mRNA#37	Madrid	Male	70	IB	T2aN0M0
mRNA#38	Madrid	Female	80	IB	T1bN0M0
mRNA#39	Madrid	Female	69	IB	T1bN0M0
mRNA#40	Madrid	Male	40	IB	T2aN0M0
mRNA#41	Madrid	Male	86	IB	T2aN0M0
mRNA#42	Madrid	Female	37	IB	T2aN0M0
mRNA#43	Madrid	Female	51	IIA	T2bN0M0
mRNA#44	Madrid	Male	79	IIA	T3aN0M0
mRNA#45	AVAST-M	Female	61	IIB	NK
mRNA#46	AVAST-M	Male	47	IIB	NK
mRNA#47	AVAST-M	Male	72	IIB	NK
mRNA#48	AVAST-M	Male	38	IIB	NK
mRNA#49	AVAST-M	Female	36	IIB	NK
mRNA#50	AVAST-M	Female	48	IIB	NK
mRNA#51	AVAST-M	Male	58	IIB	NK
mRNA#52	AVAST-M	Male	40	IIB	NK
mRNA#53	AVAST-M	Male	43	IIB	NK
mRNA#54	AVAST-M	Female	54	IIB	NK
mRNA#55	Madrid	Male	75	IIB	T4aN0M0
mRNA#56	Madrid	Female	64	IIB	T3bN0M0
mRNA#57	Madrid	Female	85	IIB	T3bN0M0
mRNA#58	Madrid	Female	79	IIB	T3bN0M0

mRNA#59	AVAST-M	Female	60	IIC	NK
mRNA#60	AVAST-M	Female	45	IIC	NK
mRNA#61	AVAST-M	Female	56	IIC	NK
mRNA#62	AVAST-M	Female	65	IIC	NK
mRNA#63	AVAST-M	Male	70	IIC	NK
mRNA#64	AVAST-M	Male	67	IIC	NK
mRNA#65	AVAST-M	Female	57	IIC	NK
mRNA#66	AVAST-M	Female	54	IIC	NK
mRNA#67	AVAST-M	Female	62	IIC	NK
mRNA#68	AVAST-M	Male	52	IIC	NK
mRNA#69	AVAST-M	Male	63	IIC	NK
mRNA#70	Madrid	Female	84	IIC	T4bN0M0
mRNA#71	Madrid	Female	46	IIC	T4bN0M0
mRNA#72	Madrid	Male	58	IIC	T4bN0M0
mRNA#73	Madrid	Female	84	IIC	T4bN0M0
mRNA#74	AVAST-M	Female	40	IIIA	NK
mRNA#75	AVAST-M	Female	40	IIIA	NK
mRNA#76	AVAST-M	Female	43	IIIA	NK
mRNA#77	AVAST-M	Male	60	IIIA	NK
mRNA#78	AVAST-M	Male	35	IIIA	NK
mRNA#79	AVAST-M	Male	54	IIIA	NK
mRNA#80	AVAST-M	Male	65	IIIA	NK
mRNA#81	AVAST-M	Female	37	IIIA	NK
mRNA#82	AVAST-M	Male	59	IIIA	NK
mRNA#83	AVAST-M	Female	37	IIIA	NK
mRNA#84	AVAST-M	Male	37	IIIA	NK
mRNA#85	AVAST-M	Male	24	IIIA	NK
mRNA#86	AVAST-M	Male	62	IIIA	NK
mRNA#87	AVAST-M	Male	65	IIIA	NK
mRNA#88	AVAST-M	Male	56	IIIA	NK
mRNA#89	AVAST-M	Male	61	IIIA	NK
mRNA#90	AVAST-M	Female	37	IIIA	NK
mRNA#91	Madrid	Male	69	IIIA	NK
mRNA#92	AVAST-M	Female	45	IIIB	NK
mRNA#93	AVAST-M	Female	51	IIIB	NK
mRNA#94	AVAST-M	Male	62	IIIB	NK
mRNA#95	AVAST-M	Female	70	IIIB	NK
mRNA#96	AVAST-M	Female	54	IIIB	NK
mRNA#97	AVAST-M	Female	65	IIIB	NK
mRNA#98	AVAST-M	Female	41	IIIB	NK
mRNA#99	AVAST-M	Male	66	IIIB	NK
mRNA#100	AVAST-M	Female	72	IIIB	NK
mRNA#101	AVAST-M	Male	55	IIIB	NK
mRNA#102	AVAST-M	Male	43	IIIB	NK
mRNA#103	AVAST-M	Male	57	IIIB	NK
mRNA#104	AVAST-M	Female	65	IIIB	NK
mRNA#105	AVAST-M	Female	29	IIIB	NK

mRNA#106	AVAST-M	Female	49	IIIB	NK
mRNA#107	AVAST-M	Male	61	IIIB	NK
mRNA#108	AVAST-M	Male	59	IIIB	NK
mRNA#109	AVAST-M	Male	61	IIIB	NK
mRNA#110	AVAST-M	Male	50	IIIB	NK
mRNA#111	AVAST-M	Male	23	IIIB	NK
mRNA#112	AVAST-M	Female	62	IIIB	NK
mRNA#113	Madrid	Male	88	IIIB	T1aN2cM0
mRNA#114	Madrid	Female	70	IIIB	T1aN2cM0
mRNA#115	Oxford	NK	NK	IIIB-C	NK
mRNA#116	AVAST-M	Female	33	IIIC	NK
mRNA#117	AVAST-M	Female	76	IIIC	NK
mRNA#118	AVAST-M	Male	55	IIIC	NK
mRNA#119	AVAST-M	Male	63	IIIC	NK
mRNA#120	AVAST-M	Male	48	IIIC	NK
mRNA#121	AVAST-M	Male	53	IIIC	NK
mRNA#122	AVAST-M	Male	50	IIIC	NK
mRNA#123	AVAST-M	Female	46	IIIC	NK
mRNA#124	AVAST-M	Male	27	IIIC	NK
mRNA#125	AVAST-M	Female	80	IIIC	NK
mRNA#126	AVAST-M	Female	73	IIIC	NK
mRNA#127	AVAST-M	Female	64	IIIC	NK
mRNA#128	AVAST-M	Female	53	IIIC	NK
mRNA#129	AVAST-M	Female	41	IIIC	NK
mRNA#130	AVAST-M	Male	58	IIIC	NK
mRNA#131	AVAST-M	Male	59	IIIC	NK
mRNA#132	Madrid	Female	81	IIIC	T4bN3M0
mRNA#133	Madrid	Male	85	IIIC	T3bN3M0
mRNA#134	Madrid	Female	80	IIIC	T3bN2cM0
mRNA#135	Madrid	Male	74	IIIC	T2bN3M0
mRNA#136	Madrid	Male	70	IV	T4aN2M1
mRNA#137	Madrid	Female	55	IV	T4aN2M1
mRNA#138	Madrid	Male	46	IV	TxN3M1
mRNA#139	Madrid	Female	71	IV	TxN2cM1
mRNA#140	Madrid	Male	58	IV	Metástasis melanoma
mRNA#141	Madrid	Female	55	IV	T3aN0M1
mRNA#142	Madrid	Male	61	IV	T4aN3M1
mRNA#143	Oxford	NK	NK	IV	NK

Table S3. Clinical details of patients used in miRNA validation cohort. NK—not known.

ID	Cohort	Gender	Age	Stage	TNM
Cont#1	Biobanco	Female	59	Control	-
Cont#2	Biobanco	Male	55	Control	-
Cont#3	Biobanco	Female	65	Control	-
Cont#4	Biobanco	Female	68	Control	-
Cont#5	Biobanco	Female	64	Control	-
Cont#6	Biobanco	Male	60	Control	-

Cont#7	Biobanco	Male	57	Control	-
Cont#8	Biobanco	Male	51	Control	-
Cont#9	Biobanco	Male	51	Control	-
Cont#10	Biobanco	Male	56	Control	-
Cont#11	Biobanco	Male	62	Control	-
Cont#12	Biobanco	Female	56	Control	-
Cont#13	Biobanco	Female	60	Control	-
Cont#14	Biobanco	Female	58	Control	-
Cont#15	Biobanco	NK		Control	-
Cont#16	Biobanco	NK		Control	-
Cont#17	Biobanco	Female	64	Control	-
Cont#18	Biobanco	Female	54	Control	-
Cont#19	Biobanco	Male	53	Control	-
Cont#20	Biobanco	Female	55	Control	-
Cont#21	Biobanco	Male	59	Control	-
Cont#22	Biobanco	Female	57	Control	-
Cont#23	Biobanco	Male	58	Control	-
Cont#24	Biobanco	Female	60	Control	-
Cont#25	Biobanco	Female	60	Control	-
Cont#26	Biobanco	Female	56	Control	-
Cont#27	Biobanco	Male	55	Control	-
Cont#28	Biobanco	Male	58	Control	-
miRNA#1	San Sebastián	Male	66	0	pT1sN0M0
miRNA#2	San Sebastián	Female	41	0	pTisN0M0
miRNA#3	San Sebastián	Female	45	0	pTisN0M0
miRNA#4	San Sebastián	Female	51	0	pTisN0M0
miRNA#5	San Sebastián	Female	62	0	pT1sN0M0
miRNA#6	Madrid	Male	66	0	Léntigo maligno
miRNA#7	Madrid	NK	75	0	TisN0M0
miRNA#8	Madrid	Male	52	0	Léntigo maligno. In situ
miRNA#9	Madrid	NK	52	0	Léntigo maligno. In situ
miRNA#10	Madrid	Female	84	0	Léntigo maligno. In situ
miRNA#11	Madrid	Male	43	0	Léntigo maligno. In situ
miRNA#12	Madrid	Female	33	0	TisN0M0
miRNA#13	Madrid	NK	61	0	TisN0M0
miRNA#14	Madrid	NK	43	0	TisN0M0
miRNA#15	San Sebastián	Female	55	0	pT1sN0M0
miRNA#16	San Sebastián	Female	77	0	pT1a Melanoma in situ
miRNA#17	San Sebastián	Female	47	0	pT1sN0M0
miRNA#18	San Sebastián	Female	15	0	pT1sN0M0
miRNA#19	San Sebastián	Female	43	0	pT1sN0M0
miRNA#20	San Sebastián	Female	59	0	pT1sN0M0
miRNA#21	San Sebastián	Female	49	0	pT1sN0M0
miRNA#22	San Sebastián	Female	51	0	pT1s
miRNA#23	San Sebastián	Female	65	0	pT1sN0M0
miRNA#24	San Sebastián	Female	51	0	pT1sN0M0
miRNA#25	San Sebastián	Male	72	0	pT1sN0M0

miRNA#26	San Sebastián	Female	52	0	pT1sN0M0
miRNA#27	San Sebastián	Female	48	0	pT1sN0M0
miRNA#28	San Sebastián	Female	40	0	pT1sN0M0
miRNA#29	San Sebastián	Male	51	0	pT1sN0M0
miRNA#30	San Sebastián	Female	43	IA	pT1aN0M0
miRNA#31	San Sebastián	Female	48	IA	pT1aN0M0
miRNA#32	San Sebastián	Female	33	IA	pT1aN0M0
miRNA#33	San Sebastián	Female	48	IA	pT1aN0M0
miRNA#34	San Sebastián	Male	44	IA	pT1aN0M0
miRNA#35	San Sebastián	Male	31	IA	pT1aN0M0
miRNA#36	Madrid	Female	37	IA	T1aN0M0
miRNA#37	Madrid	NK	75	IA	T1aN0M0
miRNA#38	Madrid	NK	NK	IA	T1aN0M0
miRNA#39	Madrid	Male	57	IA	T1aN0M0
miRNA#40	Madrid	NK	80	IA	T1aN0M0
miRNA#41	San Sebastián	Male	67	IB	PT2aN0M0
miRNA#42	Madrid	Male	86	IB	T2aN0M0
miRNA#43	Madrid	NK	67	IB	T2aN0M0
miRNA#44	Madrid	NK	60	IB	T2aN0M0
miRNA#45	Madrid	Female	37	IB	T2aN0M0
miRNA#46	Madrid	NK	76	IB	T1bN0M0
miRNA#47	San Sebastián	Female	68	IIA	pT2bN0M0
miRNA#48	Madrid	NK	71	IIA	T3aN0M0
miRNA#49	Madrid	Male	79	IIA	T3aN0M0
miRNA#50	San Sebastián	Female	71	IIA	pT3a
miRNA#51	San Sebastián	Female	69	IIA	pT2bN0
miRNA#52	San Sebastián	Male	48	IIB	pT4N0M0
miRNA#53	Madrid	Female	85	IIB	T3bN0M0
miRNA#54	Madrid	Female	79	IIB	T3bN0M0
miRNA#55	San Sebastián	Female	83	IIB	pT4aN0M0.
miRNA#56	San Sebastián	Male	51	IIB	pT3bN0M0
miRNA#57	Madrid	NK	64	IIC	T4bN0M0
miRNA#58	Madrid	Female	84	IIC	T4bN0M0
miRNA#59	Madrid	NK	87	IIC	T4bN0M0
miRNA#60	San Sebastián	Female	53	IIC	t4bn0M0
miRNA#61	San Sebastián	Male	62	IIC	t4bn0M0
miRNA#62	San Sebastián	NK	47	IIC	pT4bN0M0
miRNA#63	San Sebastián	Female	55	III	TxN1M0
miRNA#64	San Sebastián	Female	20	IIIA	pT3N1aM0
miRNA#65	San Sebastián	Male	62	IIIA	pT3aN2aM0
miRNA#66	San Sebastián	Male	42	IIIA	pT2N1aM0
miRNA#67	Madrid	NK	54	IIIA	T4aN2aM0
miRNA#68	San Sebastián	Female	58	IIIA	T2aN1aM0
miRNA#69	San Sebastián	Female	40	IIIA	-
miRNA#70	San Sebastián	Male	45	IIIA	txn2m0
miRNA#71	San Sebastián	Male	57	IIIA	t4an1am0
miRNA#72	San Sebastián	Female	52	IIIA	t3an1m0

miRNA#73	AVAST-M	Male	53	IIIA	
miRNA#74	San Sebastián	Female	41	IIIA	pT3bN1cM0
miRNA#75	San Sebastián	Female	65	IIIB	pT2bN2aM0
miRNA#76	San Sebastián	Female	62	IIIB	T3bN2cM0
miRNA#77	San Sebastián	Female	38	IIIB	T2aN2aM0
miRNA#78	San Sebastián	Female	60	IIIB	T2aN1bM0
miRNA#79	AVAST-M	Female	55	IIIB	
miRNA#80	AVAST-M	Female	60	IIIB	
miRNA#81	AVAST-M	Male	33	IIIB	
miRNA#82	AVAST-M	Male	48	IIIB	
miRNA#83	AVAST-M	Female	46	IIIB	
miRNA#84	San Sebastián	Female	62	IIIB	pT3N2aM0
miRNA#85	San Sebastián	Male	69	IIIC	pT3aN3M0
miRNA#86	Madrid	NK	44	IIIC	T2bN3M0
miRNA#87	Madrid	Male	74	IIIC	T2bN3M0
miRNA#88	San Sebastián	Female	41	IIIC	pT3aN3M0
miRNA#89	Madrid	Female	80	IIIC	T3bN2cM0
miRNA#90	Madrid	Female	71	IV	TxN2cM1
miRNA#91	Madrid	Male	61	IV	T4aN3M1
miRNA#92	Madrid	Female	55	IV	T3aN0M1
miRNA#93	Madrid	NK		IV	metastatic
miRNA#94	San Sebastián	Male	59	IV	t4bn0m1a
miRNA#95	San Sebastián	Female	48	IV	E.IV (M1b)
miRNA#96	Madrid	Male	58	IV	metastatic

Table S4. Clinical details of patients used in yRNA validation cohort. NK—not known.

ID	Origin	Gender	Age	Stage	TNM
Cont#1		Male	53	Control	-
Cont#2		Male	51	Control	-
Cont#3		Male	56	Control	-
Cont#4		Male	62	Control	-
Cont#5		Female	56	Control	-
Cont#6		Female	60	Control	-
Cont#7		Female	60	Control	-
Cont#8		Female	58	Control	-
Cont#9		Female	64	Control	-
Cont#10		Female	54	Control	-
Cont#11		Male	53	Control	-
Cont#12		Female	55	Control	-
Cont#13		Male	59	Control	-
Cont#14		Female	57	Control	-
Cont#15		Male	58	Control	-
Cont#16		Female	60	Control	-
Cont#17		Female	60	Control	-
Cont#18		Female	56	Control	-
Cont#19		Male	55	Control	-
Cont#20		Male	58	Control	-

Cont#21	Male	60	Control	-
Cont#22	Male	53	Control	-
yRNA#1	Male	45	0	TisN0M0
yRNA#2	NK	41	0	TisN0M0
yRNA#3	NK	45	0	TisN0M0
yRNA#4	NK	51	0	TisN0M0
yRNA#5	NK	62	0	T1sN0M0
yRNA#6	Male	84	0	TisN0M0
yRNA#7	Male	56	0	TisN0M0
yRNA#8	Female	47	0	pT1sN0M0
yRNA#9	Female	15	0	pT1sN0M0
yRNA#10	Female	43	0	pT1sN0M0
yRNA#11	Female	59	0	pT1sN0M0
yRNA#12	Female	49	0	pT1sN0M0
yRNA#13	Female	51	0	pT1s
yRNA#14	Female	65	0	pT1sN0M0
yRNA#15	Female	51	0	pT1sN0M0
yRNA#16	Male	71	0	pT1sN0M0
yRNA#17	Female	52	0	pT1sN0M0
yRNA#18	Female	48	0	pT1sN0M0
yRNA#19	Female	40	0	pT1sN0M0
yRNA#20	Male	51	0	pT1sN0M0
yRNA#21	Female	39	IA	T1N0M0
yRNA#22	Female	43	IA	T1aN0M0
yRNA#23	Female	48	IA	T1aN0M0
yRNA#24	Female	33	IA	T1aN0M0
yRNA#25	Female	48	IA	T1aN0M0
yRNA#26	Male	44	IA	T1aN0M0
yRNA#27	Male	31	IA	T1aN0M0
yRNA#28	Male	16	IB	T2aN0M0
yRNA#29	Male	67	IB	T2aN0M0
yRNA#30	Male	79	IIA	T3N0M0
yRNA#31	Female	68	IIA	T2bN0M0
yRNA#32	Male	72	IIB	T4aN0M0
yRNA#33	Male	67	IIB	T4aN0M0
yRNA#34	Male	48	IIB	T4N0M0
yRNA#35	Female	46	IIC	T4bN0M0
yRNA#36	Male	58	IIC	T4bN0M0
yRNA#37	Female	84	IIC	T4bN0M0
yRNA#38	Female	55	III	NK
yRNA#39	Female	41	IIIA	T3bN1cM0
yRNA#40	Female	49	IIIA	T4N1M0
yRNA#41	Male	62	IIIA	T3aN2aM0
yRNA#42	Male	42	IIIA	T2N1aM0
yRNA#43	Male	69	IIIA	T4aN1M0
yRNA#44	Female	40	IIIA	NK
yRNA#45	Female	60	IIIA	pT2aN1aM0

yRNA#46	Female	40	IIIB	pT2aN2aM0
yRNA#47	Female	65	IIIB	T2bN2aM0
yRNA#48	Male	88	IIIB	T1aN2cM0
yRNA#49	Female	62	IIIB	pT3bN2c
yRNA#50	Female	60	IIIB	pT2aN1bM0
yRNA#51	Male	69	IIIC	T3bN2cM0
yRNA#52	Male	69	IIIC	T3aN3M0
yRNA#53	Female	81	IIIC	T4bN3M0
yRNA#54	Female	80	IIIC	T3bN2cM0
yRNA#55	Male	61	IV	T4aN3M1
yRNA#56	Female	71	IV	TxN2cM1
yRNA#57	Female	55	IV	T3aN0M1
yRNA#58	Male	58	IV	Metastasis

Table S5. Characteristics of selected differentially protein-encoding gene fragment probe sets.

probe set ID	*Gene*	*Exon (Amino acid position)**	*Length*	*Sequence*
2648985	***ATM***	***1* (63-109)**	***143***	*ATTTTTACAGAAATATATTCAGAAAGAAACAGAATGTCTGAGAATAGCAAAACCAAATGTATCAGCCTCAACACAAGCCTCCAGGCAGAAAAAGATGCAGGAAATCAGTAGTTTGGTCAAATACTTCATCAAATGTGCAAACAGAA*
667389	***AMFR***	***1 (59nt(5ÚTR)1-10)***	***104***	*AATTGGGACAGGCCTCCTCCAGTCAGTCGGGCACAGGGCGCAGGAGGCAGCTGAGGAAGGCAAGGGAGCGCTAGGAGGTCTGCTGCTTCTGAAGCCTCCGTTCCG*
731164	***CD109***	**5 (170-211)**	***125***	*GACCCCAAATCAAATTTGATCCAACAGTGGTTGTCACAACAAAGTGATCTTGGAGTCATTTCCAAAACTTTTCAGCTATCTTCCCATCCAATACTTGGTGACTGGTCTATTCAAGTTCAAGTGAAT*
730641	***SOS1***	***8* (798-836)**	***119***	*TTCTCAAACCACAGAGTGAGGTTGGTGGTATGTCGAATCATTTTCAGAAGATTAGGAGAGTTAATTTCTTTGTCTTCTTTTGTCCACACACTTCCAACTAATTCTGATGGCTGTACAGCT*

* positions and exon number relative to refseq transcript.

Table S6. Summary of NGS results from pooled RNA samples.

**Pool (n, μg (RNA))**	**Reads (10^6)**	**Phread score**	**Mapped (10^6)**	**Mapped sRNA* (10^6)**
Cont. (8, 2.96)	49.79	37.93	27.83 (55%)	14.38 (29%)
Stage 0 (5, 2.07)	57.91	37.89	31.94 (55%)	11.68 (20%)
Stage I/II (8, 3.45)	49.37	37.86	24.97 (51%)	13.14 (27%)
Stage III (7, 3.20)	49.15	37.87	26.54 (54%)	12.20 (25%)
Stage IV (8, 2.96)	41.45	37.89	20.69 (50%)	9.14 (23%)

* sRNA (shortRNA) was defined as sequences between 18–43 nt.

Table S7. YRNA reads from NGS. *% of total YRNA counts.

	Cont.	Stage 0	Stage I/II	Stage III	Stage IV
**RNY/RNY4 ***	1,494,240 (25.6%) /1,399,490 (24%)	1,233,516 (26.2%) /1,185,336 (25.2%)	1,141,496(25.3%) /1,085,019 (24.1%)	1,284,278 (26.4%) /1,175,099 (24.2%)	1,179,907 (27.1%) /1,120,159 (25.7%)
**RNYP/RNYP4 ***	2,833,938 (48.6%) /2,830,682 (48.5%)	2,290,363 (48.7%) /2,288,302 (48.7%)	2,190,406 (48.6%) /2,187,535 (48.5%)	2,313,285 (47.6%) /2,310,512 (47.6%)	2,113,392 (48.5%) /2,111,236 (48.4%)
**Rfam YRNA ***	1,511,724 (25.9%)	1,176,482 (25%)	1,176,210 (26.1%)	1,258,188 (25.9%)	1,065,461 (24.4%)
**Total reads**	**5,839905**	**4,700,362**	**4,508,112**	**4,855,751**	**4,358,760**

© 2019 by the authors. Submitted for possible open access publication under the terms and conditions of the Creative Commons Attribution (CC BY) license (http://creativecommons.org/licenses/by/4.0/).
